# Knockdown of GSK3β increases basal autophagy and AMPK signalling in nutrient-laden human aortic endothelial cells

**DOI:** 10.1042/BSR20160174

**Published:** 2016-09-16

**Authors:** Karen A. Weikel, José M. Cacicedo, Neil B. Ruderman, Yasuo Ido

**Affiliations:** * Department of Medicine, Boston University School of Medicine and Boston Medical Center, 650 Albany Street, Boston, MA 02118, U.S.A.

**Keywords:** AMP-activated protein kinase (AMPK), autophagy, endothelium, forkhead box protein O1 (FOXO1), glycogen synthase kinase 3β (GSK3β)

## Abstract

High concentrations of glucose and palmitate increase endothelial cell inflammation and apoptosis, events that often precede atherogenesis. They may do so by decreasing basal autophagy and AMP-activated protein kinase (AMPK) activity, although the mechanisms by which this occurs are not clear. Decreased function of the lysosome, an organelle required for autophagy and AMPK, have been associated with hyperactivity of glycogen synthase kinase 3β (GSK3β). To determine whether GSK3β affects nutrient-induced changes in autophagy and AMPK activity, we used a primary human aortic endothelial cell (HAEC) model of type 2 diabetes that we had previously characterized with impaired AMPK activity and autophagy [Weikel et al. (2015) Am. J. Phys. Cell Physiol. **308**, C249–C263]. Presently, we found that incubation of HAECs with excess nutrients (25 mM glucose and 0.4 mM palmitate) increased GSK3β activity and impaired lysosome acidification. Suppression of GSK3β in these cells by treatment with a chemical inhibitor or overexpression of kinase-dead GSK3β attenuated these lysosomal changes. Under control and excess nutrient conditions, knockdown of GSK3β increased autophagosome formation, forkhead box protein O1 (FOXO1) activity and AMPK signalling and decreased Akt signalling. Similar changes in autophagy, AMPK and Akt signalling were observed in aortas from mice treated with the GSK3β inhibitor CHIR 99021. Thus, increasing basal autophagy and AMPK activity by inhibiting GSK3β may be an effective strategy in the setting of hyperglycaemia and dyslipidaemia for restoring endothelial cell health and reducing atherogenesis.

## Abstract (precis)

Suppression of the enzyme glycogen synthase kinase 3β (GSK3β) increases both the turnover of damaged cellular material and the activity of the enzyme AMP-activated protein kinase (AMPK) to potentially attenuate the damage inflicted by excess sugar and fat on blood vessels.

## INTRODUCTION

During hyperglycaemia and dyslipidaemia, dysregulation of macroautophagy [1] (hereafter referred to as ‘autophagy’) and AMP-activated protein kinase (AMPK) [1–3] is associated with endothelial cell damage and dysfunction, events that often precede atherogenesis [4,5]. Despite this, the mechanisms by which high concentrations of glucose and fatty acids such as palmitate alter autophagy and AMPK activity are not well-understood.

Autophagy is a process by which a cell digests long-lived proteins and organelles to either synthesize new ones or generate fuel. These proteins and/or organelles are recruited to a double-membrane vesicle called an autophagosome. The autophagosome then fuses with the lysosome to create an autolysosome, where its cargo can be degraded by proteases. Autophagy can be induced by conditions such as starvation or inflammation, but also occurs constitutively under non-starvation conditions in what is referred to as basal autophagy [6], a process critical for the maintenance of cellular homoeostasis in the vasculature. Compared with wild-type mice, basal autophagy is impaired in the aorta of ApoE-null mice [7], a model of atherosclerosis, and its impairment in macrophages is associated with increased plaque development [7–9]. Additional evidence illustrating the important role of autophagy in vascular health can be found in studies using high-fat fed rabbits, in which activation of macrophage autophagy through the use of mammalian target of rapamycin complex 1 (mTORC1) inhibitors has been shown to slow progression of atherosclerosis and stabilize plaques [10–12]. Autophagic and lysosomal dysregulation has also been observed in human umbilical vein endothelial cells (HUVECs) treated with oxidized LDL [13], a contributor to plaque development. In a human aortic endothelial cell (HAEC) culture model that we developed to mimic the diabetic milieu during early atherogenesis, we observed impairment of autophagosome formation and lysosome enzyme activity [1]. In this model, chronic incubation of the cells in media containing 25 mM glucose, followed by an acute exposure to 0.4 mM palmitate, elicited several pro-atherogenic events in addition to impaired basal autophagy including inflammation, apoptosis and reduced activity of AMPK [1]. Activation of AMPK [14] and restoration of basal autophagy with rapamycin [1] attenuated the effects of excess nutrients on inflammation and apoptosis. Interestingly, activation of AMPK, an autophagy inducer under low-nutrient conditions [15–17], did not induce autophagy in HAECs in the presence of excess nutrients [1]. To gain insight into potential therapeutic targets for restoring autophagy under excess nutrient conditions, we carried out studies to elucidate the mechanism(s) by which high concentrations of glucose and palmitate impair basal autophagy in HAECs.

A focus of these studies was glycogen synthase kinase 3β (GSK3β), an enzyme whose activity is increased in both humans and experimental animals with diabetes [18–20]. GSK3β has been shown to phosphorylate AMPK at Thr^479^ thus inhibiting its activity in HEK293 cells, mouse embryonic fibroblasts (MEFs), neutrophils and macrophages [21,22]. It can also inhibit autophagy by several possible mechanisms including diminution of lysosome acidification (as suggested by studies in SHSY-5Y [23] and MCF-7 cells [24]), decreased nuclear translocation of transcription factor EB (TFEB), a transcription factor for autophagy and lysosomal genes (as suggested by studies in HEK293 and human pancreatic cancer cells) [25], and perhaps under certain conditions by suppressing the activity of AMPK [21,22,26] or activating mTORC1 [24]. If and how GSK3β affects basal autophagy in HAECs exposed to excess nutrients has not been studied.

For all of the above reasons, we first evaluated whether the diabetic milieu alters GSK3β activity in HAECs. We then investigated how inhibition of GSK3β activity affected lysosome acidification and autophagosome formation in HAECs exposed to excess nutrients. Finally, we assessed changes in autophagy- and AMPK-related signalling in nutrient-laden HAECs subjected to GSK3β inhibition and in aortas of mice treated with the GSK3β inhibitor, CHIR 99021.

## MATERIALS AND METHODS

### Materials

HAECs (catalogue # cc-2535) and EGM-2 media (catalogue # cc-3162) were purchased from Lonza. Bafilomycin (catalogue # B1793), DMSO (catalogue # D2438) and Akt 1/2 kinase inhibitor (Akt inhibitor VIII or Akti) (catalogue # A6730) were purchased from Sigma, CHIR 99021 from Tocris Bioscience and palmitate from Nu-Chek Prep. Antibodies raised against LC3-II (catalogue # 3868S), phosphorylated (Thr^172^) AMPK(α1/α2) (catalogue # 2531S), phosphorylated (Ser^485^) AMPK α1 (catalogue # 4185), phosphorylated (Ser^79^) ACC (catalogue # 3661S), phosphorylated (Ser^2448^) mTOR (catalogue # 2971S), total mTOR (catalogue # 2972S), phosphorylated (Ser^9^) GSK3β (catalogue # 5558), total GSK3β (catalogue # 12456), phosphorylated (Ser^641^) glycogen synthase (catalogue # 3891), phosphorylated (Ser^473^) Akt1 (catalogue # 9018P), total Akt (catalogue # 9272), phosphorylated (Thr^24^) FOXO1 (catalogue # 2599S) and LAMP1 (catalogue # 9091P) were purchased from Cell Signaling Technology. Antibody raised against phosphorylated (Thr^308^) Akt was purchased from Upstate Biotechnologies (now Millipore). Antibody raised against total AMPKα1 (catalogue # 3694-1) was purchased from Epitomics and against β-actin (catalogue # A4700) from Sigma. Antibody raised against Hsp90α/β (catalogue # sc-7947) and GAPDH (catalogue # sc-25778) from Santa Cruz Biotechnology. Secondary peroxidase conjugated anti-rabbit (catalogue # NA934V) antibody was obtained from GE Healthcare.

### Cell culture

HAECs were grown in EGM-2 media containing 2% FBS on Primaria culture dishes (reference no. 353803) (BD Biosciences). Excess nutrient conditions were implemented as described previously [1]. For hyperglycaemic conditions, cells were grown and passaged in EGM-2 media supplemented with D-glucose (catalogue # G-7021) (Sigma) to a final concentration of 25 mM. The cells were then incubated in hyperglycaemic media beginning in passage 3 and when they reached 85% confluence, were split in hyperglycaemic media for passage 4. This protocol for maintaining and splitting the cells in hyperglycaemic media was repeated for future passages. For both normoglycaemic and hyperglycaemic cells, experiments began in passage 5 and were performed when cells were 85–95% confluent. At this time, media was changed to DMEM (Life Technologies) (catalogue # 31600-034) supplemented with 5% FBS containing either BSA or BSA-palmitate conjugates, as described previously [1,27]. Briefly, BSA was used both to stabilize the insoluble fatty acids and to transport them to the cell. Control-treated cells were exposed to media containing 0.5 mM BSA, and fatty acid-treated cells to media containing BSA conjugated to 0.4 mM palmitate at a 3:1 FFA:BSA molar ratio. Cells were exposed to BSA or BSA-palmitate conjugates in DMEM for 6 h prior to harvesting for analysis. For experiments that included bafilomycin or Akt inhibitor VIII (Akti), they were added to the cells 30 min prior to DMEM incubation which took place over the next 6 h. For experiments using CHIR 99021, it was added to the cells 17 h prior to DMEM incubation which took place over the next 6 h.

### Recombinant lentivirus preparation

The recombinant lentivirus plasmid expressing small-hairpin RNA targeting GSK3β (ggtcacgtttggaaagaat) was created as published previously [28]. The recombinant lentivirus plasmid expressing the kinase-dead GSK3β mutant (K85A GSK3β) was created as follows. The coding sequence of human GSK3β (BC000251, from ATCC) was subcloned into a pENTR1A vector (Invitrogen) and site-specific mutation was introduced by PCR using mutation primers (forward TCAGGAGAACTGGTCGCCATCGCGAAAGTATTGCAGGACAAGAGA, re-verse TCTCTTGTCCTGCAATACTTTCGCGATGGCGACCAGTTCTCCTGA). After confirming the mutation by sequence analysis, the mutated coding sequence was transferred by LR reaction (Invitrogen) to a home-made lentivirus vector in which the CMV promoter expresses the targeting sequence. The recombinant lentivirus plasmid expressing GFP-LC3 (Addgene #22418) was created in a home-made Gateway compatible lentivector after subcloning it into a pENTR1A vector and lentivirus produced as described previously [28].

### Western blotting

Cells were harvested and lysed in a Triton-based buffer containing 20 mM Tris pH 7.5, 150 mM NaCl, 1 mM EDTA, 1 mM EGTA, 0.1% SDS, 1% Triton X-100, 2.5 mM sodium pyrophosphate, 1 mM β-glycerophosphate, 1 mM sodium orthovanadate and 1 μg/ml leupeptin. Following brief sonication, lysates were centrifuged at 16,300 ***g*** for 10 min at 4°C. The supernatants were stored at −80°C for future analysis. Proteins were separated using either 4–12% Bis–tris gradient or 14% Tris-glycine gels (Life Technologies) and transferred to PVDF membranes. After blocking for at least 1 h at room temperature with non-fat milk, membranes were incubated in primary antibody overnight. Following washing in TBST, membranes were incubated in peroxidase conjugated anti-rabbit antibody and bands were visualized on film using SuperSignalWest Pico (catalogue # 1856135 and 1856136) or Femto (catalogue # 34095) chemiluminescent substrate (Thermo Scientific). Densitometric analyses were carried out using Scion Image software and presented after adjusting for loading controls, GAPDH and β-actin, as used previously [1]. We observed similar results between those analyses based upon GAPDH and those based upon β-actin.

### Lysosome acidification

HAECs were treated with Lysotracker Red DND-99 (Life Technologies) (catalogue # L-7528) according to the manufacturer's instructions. Briefly, following incubation in control or excess nutrient conditions, cells were incubated with lysotracker dye for 30 min. Cells were then washed and fluorescence was visualized on a Nikon TE-200 Eclipse Inverted microscope. After capturing images they were quantified with ImageJ (NIH). For each image, fluorescence levels were adjusted for the number of cells present.

### LAMP1 immunohistochemistry

HAECs were exposed to either control or excess nutrient conditions as described above and fixed in 0.4% formalin. Antigen retrieval was performed using citrate buffer. In conjunction with anti-LAMP1 antibody (Cell Signaling Technologies) Ultratech HRP 500–600 tests (catalogue # IM2391, Beckman-Coulter) and ImmPACT AMEC Red Peroxidase HRP substrate (catalogue # SK-4285, Vector Laboratories) were used to visualize LAMP1. ImageJ (NIH) was used to quantify LAMP1 staining relative to the number of cells in each image.

### Assessment of autophagosome formation

Cells were exposed to experimental conditions as described above for 6 h, in the presence or absence of 10 nM bafilomycin. Bafilomycin-induced LC3-II protein levels, or the ratio of LC3-II/LC3-I protein, as measured by western blotting, were used as indicators of autophagosome formation. To measure LC3-II protein accumulation by fluorescence microscopy, HAECs were infected with GFP-LC3 expressing lentivirus. Three days following infection, distinct perinuclear puncta are indicative of LC3-II. Following experimental treatment, puncta fluorescence was visualized on a Nikon TE-200 Eclipse Inverted microscope and quantified with ImageJ (NIH). For each well, the percentage of GFP-LC3 infected cells that expressed distinct perinuclear puncta was multiplied by the average GFP fluorescence per cell to calculate the GFP-LC3 puncta measurement. This score was averaged across all wells of the treatment group.

### Real-time PCR

SYBR green Master Mix from Clontech Laboratories (catalogue #RR420A) was used to quantify real-time PCR product on Cepheid's Smart Cycler. The following primers were used to detect mRNAs: β-actin: 5′-TTGTAACCAACTGGGACGATATGG-3′ (sense) and 5′-GATCTTGATCTTCATGGTGCTAGG-3′ (antisense), PIK3C3: 5′-GCCACCAGTACAAAACATGGCT-3′ (sense) and 5′-TGGCCCATTCTCACTTGGTGC-3′ (antisense) and SOD2: 5′-TGTCCAAATCAGGATCCACTGC-3′ (sense) and 5′-GGCCTGACATTTTTATACTGAAGGT-3′ (antisense). mRNA levels of PIK3C3 and SOD2 were quantified using the 2(−ΔΔC(T)) method, relative to actin.

### Animal experiments

Sixteen male C57BL6/J background mice between 17 and 24 weeks of age were housed under controlled temperature (22°C), humidity (40%) and light (12 h:12 h light/dark cycle) conditions in a specific pathogen-free vivarium and provided food and water *ad libitum*. All experimental procedures were approved by the Institutional Animal Care and Use Committee. For 3 consecutive days, mice were injected intraperitoneally with either 7.5 mg/kg per day CHIR 99021 (45% saline, 45% PEG400, 10% DMSO) or saline/PEG400. Following the injection on the third day, the mice were anaesthetized with inhaled isoflurane (catalogue # 050033, Henry Schein, Dublin, OH) and killed by cervical dislocation. The thoracic aorta was harvested and periaortic fat removed with the use of a dissecting microscope, on ice. The aortas were snap-frozen in liquid nitrogen and stored at −80°C. For western blot analysis, aortas were homogenized in a tissue grinder (Kimble Chase) in a Triton-based buffer containing 20 mM Tris pH 7.5, 150 mM NaCl, 1 mM EDTA, 1 mM EGTA, 0.1% SDS, 1% Triton X-100, 2.5 mM sodium pyrophosphate, 1 mM β-glycerophosphate, 1 mM sodium orthovanadate and 1 μg/ml leupeptin. Following brief sonication, lysates were centrifuged at 16,300 ***g*** for 10 min at 4°C. The supernatants were stored at −80°C for future analysis. Protein levels were analysed in these samples by western blot. During these analyses, GAPDH was overexposed so we utilized a loading control located in a different region of the membrane, Hsp90 [29].

### Statistics

Data are presented as means±S.E.M. Unless stated otherwise, differences between treatment groups were analysed using ANOVA (GraphPad software). *P*<0.05 was considered significant. Sample sizes (*n*) are indicated in each figure legend and refer to separate cell culture wells (biological replicates) or animals. All experiments were performed independently at least twice with similar results.

## RESULTS

### High concentrations of glucose and palmitate increase GSK3β activity in HAECs

**Figure 1:**
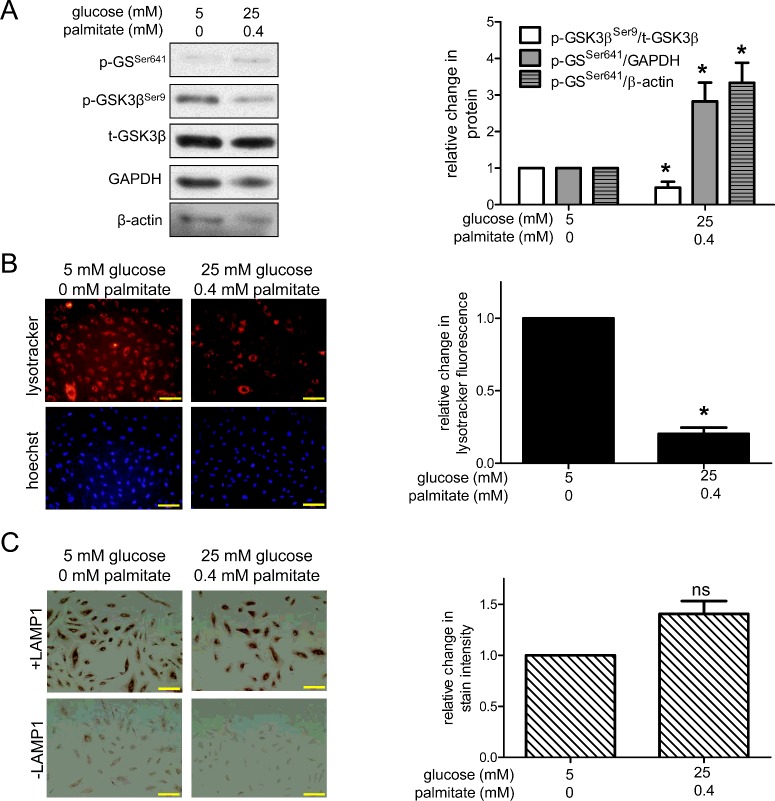
Excess nutrients increase GSK3β activity and decrease lysosome acidification (**A**) HAECs incubated in excess nutrient conditions have a decreased ratio of inhibitory p-GSK3β^Ser9^/t-GSK3β protein and increased protein levels of p-GS^Ser641^, a downstream target of GSK3β (GAPDH *n*=4; β-actin *n*=3). (**B**) Exposure to excess nutrients decreased lysotracker staining (*n*=7). (**C**) Excess nutrients did not decrease LAMP1 protein levels (*n*=5). * indicates *P*<0.05 by Student's *t* test compared with control conditions. For (**B**) and (**C**), bar=100 μm.

Increased GSK3β activity has been observed in skeletal muscle of humans with diabetes [18] and in epididymal adipose tissue [19] and hearts [20] of diabetic mice. To determine if GSK3β activity is elevated in our HAEC model of poorly-controlled type 2 diabetes, we first exposed HAECs to excess nutrient conditions (cells were passaged at least twice in media containing 25 mM glucose and then exposed to 0.4 mM palmitate for 6 h) and measured protein levels of phosphorylated GSK3β. Unlike most enzymes, GSK3β is constitutively active and is inhibited when phosphorylated at Ser^9^ [30]. Compared with control conditions (5 mM glucose, 0 mM palmitate), excess nutrient conditions decreased the ratio of phosphorylated GSK3β (p-GSK3β^Ser9^)/total GSK3β (t-GSK3β), indicating that GSK3β activity increased (Figure 1A). Glycogen synthase (GS) is a downstream target of GSK3β that is inactivated when phosphorylated at Ser^641^ by GSK3β. Consistent with the changes we observed in p-GSK3β^Ser9^/t-GSK3β, we found that excess nutrients also increased protein levels of phosphorylated GS (p-GS^Ser641^) (Figure 1A).

### Excess nutrients decrease lysosome acidification

High GSK3β activity has been reported to inhibit autophagy by interfering with lysosome acidification [23,24]. Lysosomes fuse with autophagosomes to form autolysosomes [31] and require a low pH to degrade autophagic substrates using proteases such as cathepsins [32]. Overexpression of GSK3β in MCF-7 cells [24] and SHSY-5Y cells [23] impairs lysosome acidification and chemical inhibition of GSK3β in HEK293 cells increases lysosome acidification [25]. Since excess nutrients increased GSK3β activity in our model (Figure 1A), decreased lysosome acidification could contribute to the decrease in cathepsin L activity and overall impairment of autophagic flux that we reported previously [1]. We measured the effect of excess nutrients on lysosome acidification using lysotracker red dye, which stains acidic organelles such as lysosomes and autolysosomes [33]. Indeed, excess nutrients decreased lysotracker staining (Figure 1B), suggesting that fewer of these acidic vesicles were present. To determine if this loss of lysotracker staining was due to decreased acidification or a decrease in the number of these vesicles, we used immunohistochemistry to evaluate protein expression of LAMP1, a protein that resides on the lysosomal membrane. As shown in Figure 1(C), excess nutrients did not decrease LAMP1 protein expression, indicating that excess nutrients did not decrease the number of lysosomes in HAECs, but decreased their acidification.

### Inhibition of GSK3β activity attenuates excess nutrient-induced suppression of lysosome acidification

**Figure 2:**
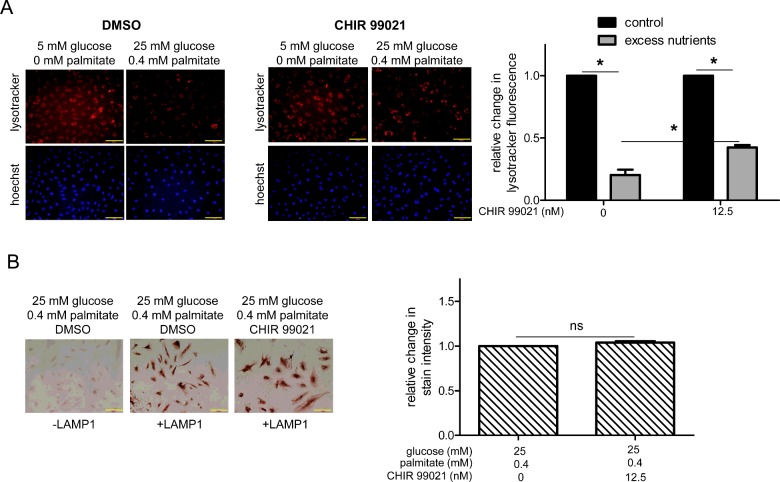
Reduction of GSK3β activity with CHIR 99021 attenuates the effects of excess nutrients on lysosome acidification (**A**) Compared with excess nutrient-exposed HAECs treated with vehicle alone (DMSO), those incubated with 12.5 nM CHIR 99021 for 23 h had more lysotracker staining. Representative images are shown adjacent to graph. * indicates *P*<0.05 by Student's *t* test (*n*=3). (**B**) CHIR 99021 treatment did not affect LAMP1 staining in cells incubated in excess nutrient conditions. ns indicates no significant difference (*n*=3). Quantification of fluorescence or LAMP1 is relative to the number of cells in each image. Bar=100 μm.

Thus far, we have shown that excess nutrients increase GSK3β activity and decrease lysosome acidification in HAECs (Figure 1). To determine the role of GSK3β activity in lysosome acidification, we decreased GSK3β activity in HAECs by infecting them with a lentivirus expressing small-hairpin RNA targeting GSK3β (shGSK3β) (Supplementary Figure S1A). We found that under both control and excess nutrient conditions, compared with cells infected with a control lentivirus (shc), cells infected with shGSK3β had more lysotracker staining (Supplementary Figure S1B). We also decreased GSK3β activity in HAECs by infecting them with a lentivirus expressing a kinase-dead mutant of GSK3β, K85A GSK3β (Supplementary Figure S2A). Compared with shc-infected cells, there was no change in lysotracker staining in K85A GSK3β-infected cells under control conditions and a small decrease in lysotracker staining under excess nutrient conditions (Supplementary Figure S2B). This decrease in excess nutrient conditions was smaller than that of non-infected cells, as shown in Figure 1(B). Although the shGSK3β and K85A GSK3β lentiviruses did not have the same effect on lysotracker staining in excess nutrient-exposed HAECs, both showed more lysotracker staining than cells with full GSK3β activity in this condition. To further evaluate the effect of GSK3β inhibition on lysosome acidification, we treated cells with CHIR 99021 [34], an ATP-competitive inhibitor, and measured excess nutrient-induced changes in lysotracker staining. Using a dose which inhibits GSK3β in excess nutrient conditions (Supplementary Figure S3), we found that CHIR 99021 treatment attenuated the effect of excess nutrients on lysotracker staining (Figure 2A). CHIR 99021 did not affect LAMP1 protein levels (Figure 2B), indicating that its increase in lysotracker staining was due to increased acidification rather than lysosome number.

To gain some insight regarding the effects of GSK3β hyperactivation on lysosome acidification, we increased GSK3β activity by treating HAECs with Akt 1/2 kinase inhibitor VIII (Akti). Since Akt is an upstream kinase that inhibits GSK3β, Akti treatment increased GSK3β activity, as indicated by a decreased ratio of p-GSK3β^Ser9^/t-GSK3β (Supplementary Figure S4A). Under both control (Supplementary Figure S4B) and excess nutrient conditions (Supplementary Figure S4C), Akti treatment decreased lysotracker staining, indicating that similar to MCF-7 cells [24] and SHSY-5Y cells [23], increased GSK3β activity in HAECs can decrease lysosome acidification. Besides activating GSK3β, Akt inhibition could decrease lysotracker staining by inhibiting mTORC1 and thus decreasing nuclear localization of TFEB, a regulator of lysosome biogenesis [35]. However, since excess nutrients did not alter LAMP1 expression (Figure 1C), it is unlikely that the excess nutrient-induced hyperactivation of GSK3β activity that we are studying affects lysosome biogenesis and TFEB.

Collectively, these data suggest that in HAECs, activation of GSK3β may be involved in excess nutrient-induced decreases in lysosome acidification.

### Inhibition of GSK3β activity increases autophagosome formation in HAECs under control and excess nutrients conditions

In addition to an acidic lysosome, efficient autophagic flux requires adequate autophagosome formation [6]. Autophagosomes can be quantified indirectly by measuring the protein levels of microtubule-associated protein light chain 3 (LC3), a membrane-bound component of the autophagosomal membrane [36]. LC3 is present in the cytosol as LC3-I, but upon conjugation with phosphatidylethanolamine it is incorporated into the developing autophagosome membrane as LC3-II. Protein levels of LC3-II, or the LC3-II/LC3-I ratio are surrogate indicators of autophagosome abundance and formation. LC3-II levels can be assessed by infecting cells with a virus expressing GFP-LC3 and monitoring fluorescence of discrete puncta. They can also be evaluated by western blot, as can the ratio of LC3-II/LC3-I. Since LC3-II is degraded by the lysosome, we performed these measurements in the presence of the lysosomal inhibitor bafilomycin A1 (hereafter referred to as simply bafilomycin) to prevent LC3-II degradation [37,38]. Thus, treatment with bafilomycin causes an accumulation of LC3-II protein. A decrease in bafilomycin-induced LC3-II protein accumulation would suggest that autophagosome formation is diminished.

**Figure 3:**
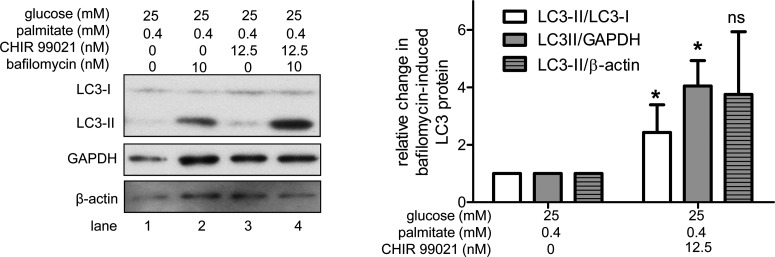
CHIR 99021 treatment increases bafilomycin-induced protein levels of the autophagosome marker LC3-II in excess nutrient conditions Treatment of excess nutrient-exposed HAECs with 12.5 nM CHIR 99021 for 23 h increased the ratio of LC3-II/GAPDH and the ratio of LC3-II/LC3-I as determined by western blot. For example, LC3-II/GAPDH in [lane 4-lane 3] > [lane 2–lane 1]. * indicates *P*<0.05 for an effect of CHIR 99021 under excess nutrient conditions by two-way ANOVA (*n*=6).

**Figure 4:**
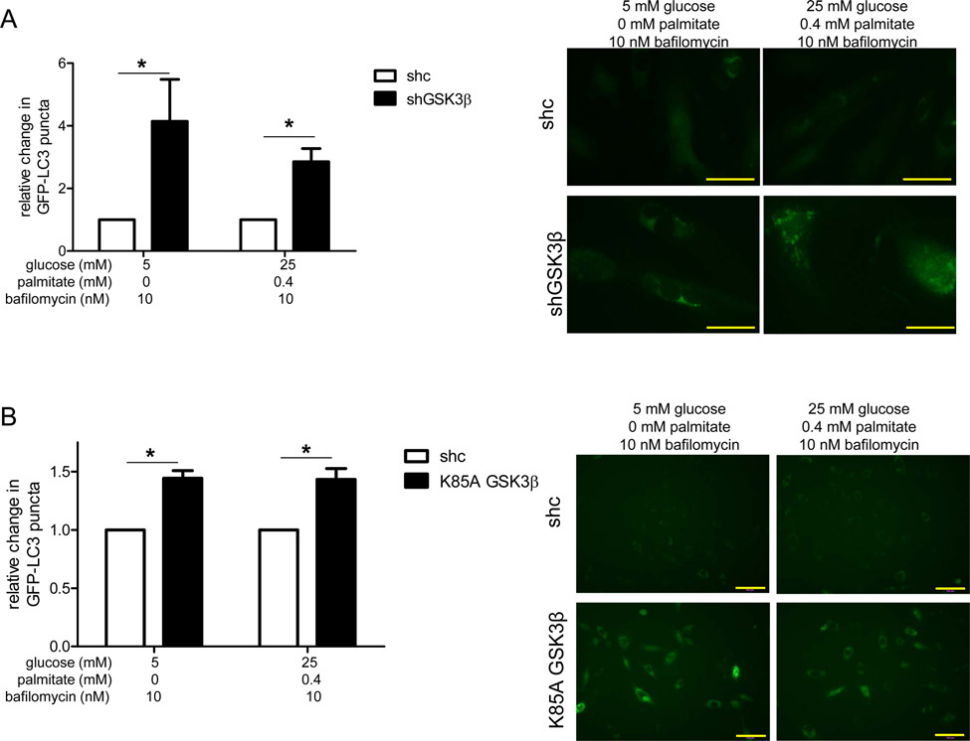
Reduction of GSK3β activity increases protein levels of LC3-II Compared with HAECs-infected with control lentivirus (shc), those infected with lentivirus reducing expression of GSK3β (shGSK3β) (**A**) or expressing kinase-dead GSK3β (K85A GSK3β) (**B**) had more GFP-LC3 puncta under both control and excess nutrient conditions. Punctate structures, rather than diffuse GFP staining is indicative of LC3-II expression. Quantification is shown relative to shc for each treatment condition, adjacent to representative images. Bar=50 μm in **A** and 100 μm in **B**. * indicates *P*<0.05 for an effect of lentivirus infection by two-way ANOVA (**A**: *n*=3; **B**: *n*=5).

Previously, we showed that excess nutrients impair autophagosome formation [1]. To explore whether GSK3β might play a role in this phenomenon, we treated excess nutrient-exposed HAECs with CHIR 99021 and measured LC3 protein levels. Under excess nutrient conditions, inhibition of GSK3β with CHIR 99021 increased the ratio of LC3-II/LC3-I and LC3-II protein levels relative to GAPDH (Figure 3). In the presence of CHIR 99021, bafilomycin-induced changes in LC3-II protein levels relative to β-actin showed a similar trend, but did not reach statistical significance. Thus, to verify the effect of GSK3β inhibition on autophagosome formation under excess nutrient conditions and extend our findings to control conditions, we used additional methods to both inhibit GSK3β activity and evaluate LC3 protein expression. We found that under both control and excess nutrient conditions, compared with cells infected with a control lentivirus (shc), cells infected with shGSK3β accumulated more LC3-II protein in the presence of bafilomycin, as measured by both GFP-LC3 puncta (Figure 4A) and western blot (Supplementary Figure S5A). We also measured LC3-II protein levels in HAECs infected with the kinase-dead mutant K85A GSK3β. Compared with cells with full GSK3β activity, K85A GSK3β-infected cells accumulated more LC3-II protein when treated with bafilomycin (Figure 4B, Supplementary Figure S5B). These data suggest that inhibition of GSK3β activity can increase autophagosome formation under both control and excess nutrient conditions.

### Knockdown of GSK3β increases Akt/AMPK-FOXO1 signalling under control and excess nutrient conditions

**Figure 5:**
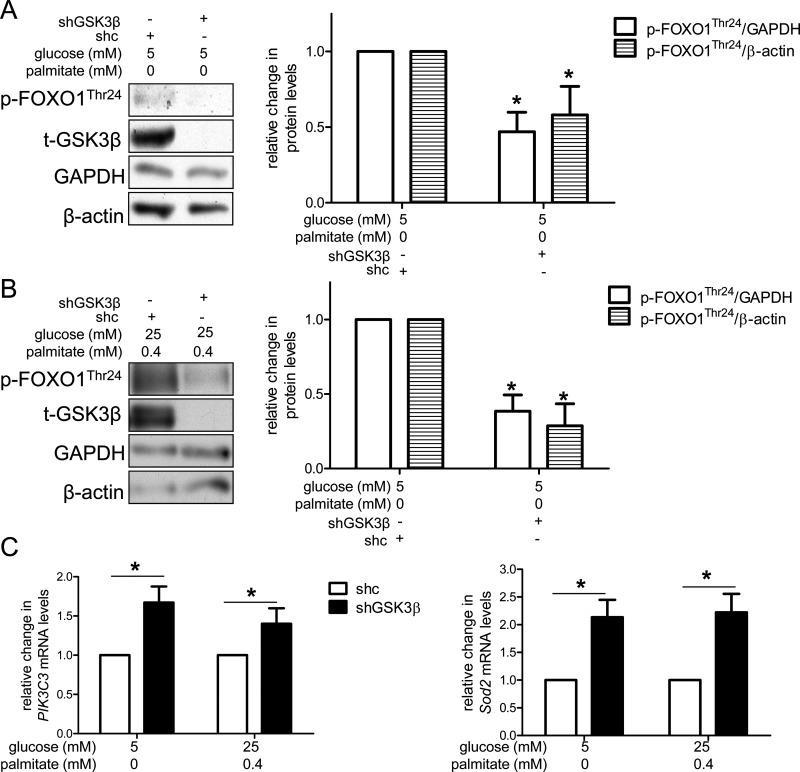
Knockdown of GSK3β protein increases FOXO1 activity (**A**) Compared with HAECs infected with control lentivirus (shc), those infected with lentivirus reducing expression of GSK3β (shGSK3β) had less p-FOXO1^Thr24^ under both control (**A**) and excess nutrient conditions (**B**). Representative western blots are shown adjacent to the graphs. (**C**) Knockdown of GSK3β (shGSK3β) increased mRNA levels of PIK3C3 (left) and SOD2 (right) compared with controls (shc). * indicates *P*<0.05 for an effect of GSK3β knockdown by two-way ANOVA (For **A** and **B**: GAPDH *n*=6; β-actin *n*=5).

To begin to address the molecular mechanism by which reduction of GSK3β activity could increase autophagosome formation, we assessed the downstream signalling changes induced by knockdown of GSK3β that could influence autophagy. mTORC1 is an established inhibitor of autophagy, and in particular, autophagosome formation [39]. Although inhibition of GSK3β increases autophagic flux by suppressing mTORC1 activity in MCF-7 cells [24], we (Supplementary Figure S6) and others [40–42] did not observe decreased mTORC1 activity in shGSK3β-infected HAECs, a discrepancy possibly due to cell type variability. Another potential modulator of autophagy is forkhead box protein O1 (FOXO1). FOXO1 has been shown to promote autophagosome formation through its transcriptional targets [43] as well as its acetylation and interaction with Atg7 [44], a protein required for autophagy. We found that knockdown of GSK3β (shGSK3β) reduced the phosphorylation of FOXO1 at Thr^24^ (p-FOXO1^Thr24^) under both control (Figure 5A) and excess nutrient conditions (Figure 5B). Reduced phosphorylation of FOXO1 at Thr^24^ has been associated with its nuclear translocation and activation [45,46]. To verify the activation of FOXO1 in our model, we measured mRNA levels of *PIK3C3* and *Sod2*, gene targets of FOXO1 that also encode for autophagy-associated proteins [47–49]. Indeed, under both control and excess nutrient conditions, knockdown of GSK3β (shGSK3β) increased mRNA levels of both of these targets (Figure 5C).

**Figure 6:**
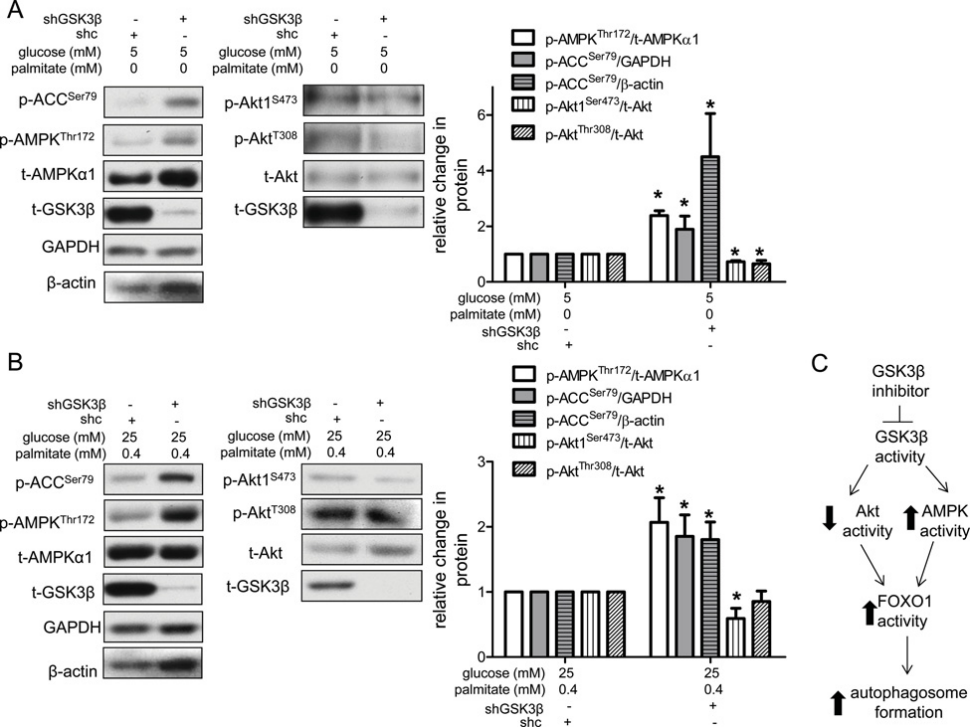
GSK3β knockdown increases AMPK signalling and decreases Akt signalling Compared with HAECs infected with control lentivirus (shc), those infected with lentivirus reducing the expression of GSK3β (shGSK3β) had an increased ratio of p-AMPK^Thr172^/t-AMPKα1 protein (*n*=5), increased p-ACC^Ser79^ (GAPDH *n*=5; β-actin *n*=4), a downstream target of AMPK and a decreased ratio of p-Akt^Ser473^/t-Akt (*n*=3) under both control (**A**) and excess nutrient conditions (**B**). shGSK3β also decreased the ratio of p-Akt^Thr308^/t-Akt (*n=5)* under control conditions (**A**). * indicates *P*<0.05 for an effect of GSK3β knockdown by ANOVA. Representative western blots are shown with densitometric analyses. Separate blots are shown for AMPK and Akt signalling due to the similarity in molecular mass of AMPK and Akt. (**C**) Proposed pathway by which suppression of GSK3β activity could increase autophagosome formation in HAECs exposed to excess nutrients.

FOXO1 is not known to be a direct target of GSK3β, but is targeted by both Akt and AMPK. When phosphorylated by Akt at Thr^24^, Ser^256^ or Ser^319^, FOXO1 activity declines [50,51], whereas phosphorylation of FOXO1 at Thr^182^, Thr^649^, Ser^544^, Ser^579^ or Ser^616^ by AMPK promotes its transcriptional activation [51–53]. Consistent with the reduction of p-FOXO1^Thr24^ protein and increase in FOXO1 target gene expression in shGSK3β-infected cells (Figure 5), we found that under control conditions knockdown of GSK3β also decreased the ratios of phosphorylated Akt (p-Akt1^Ser473^)/total Akt (t-Akt) and p-Akt^Thr308^/t-Akt, suggesting that Akt activity was decreased (Figure 6A). We also observed increased AMPK signalling. Phosphorylation of AMPK at Thr^172^ (p-AMPK^Thr172^) has been shown to increase the activity of AMPK nearly 100-fold and is often used as a surrogate marker for AMPK activity [54]. We found that compared with HAECs infected with a control lentivirus (shc), cells infected with shGSK3β had a higher ratio of p-AMPK^Thr172^/total AMPKα1 (t-AMPKα1) (Figure 6A). Acetyl-CoA carboxylase (ACC) is a substrate of AMPK that plays a role in fatty acid synthesis [55]. Consistent with the increased phosphorylation of AMPK, knockdown of GSK3β also increased phosphorylation of ACC at Ser^79^ (p-ACC^Ser79^) (Figure 6A). Similar effects of shGSK3β on Akt and AMPK signalling were observed under excess nutrient conditions (Figure 6B), although the decrease in p-Akt^Thr308^/t-Akt did not reach statistical significance. Since phosphorylation of Akt at these two sites is thought to occur via two separate kinases [56], our data suggest that PDK1, which phosphorylates Akt at Thr^308^, may be less sensitive to changes in GSK3β activity under excess nutrient conditions. Nonetheless, the effects of shGSK3β on p-AMPK^Thr172^ and p-ACC^Ser79^ are consistent with increased FOXO1 transcriptional activity (Figure 5C). Collectively, these data suggest that knockdown of GSK3β may decrease Akt activity (at least under basal conditions) to release its phosphorylation and inhibition of FOXO1. GSK3β knockdown may also increase AMPK activity, facilitating phosphorylation and activation of FOXO1 by AMPK. Such an increase in FOXO1 activity may then promote autophagosome formation (Figure 6C).

In HEK293 cells, MEFs, neutrophils and macrophages, inhibition of AMPK activity by GSK3β has been associated with phosphorylation of AMPK at both Thr^479^ and Ser^485^ [21,22]. If the converse mechanism were involved in the activation of AMPK activity by GSK3β inhibition, we might expect to find a decreased ratio of p-AMPK^Ser485^/t-AMPKα1. However, under both control and excess nutrient conditions, we did not observe any changes in this phosphorylation site (Supplementary Figure S7) upon knockdown of GSK3β.

### CHIR 99021 alters autophagy and AMPK/Akt markers *in vivo*

**Figure 7:**
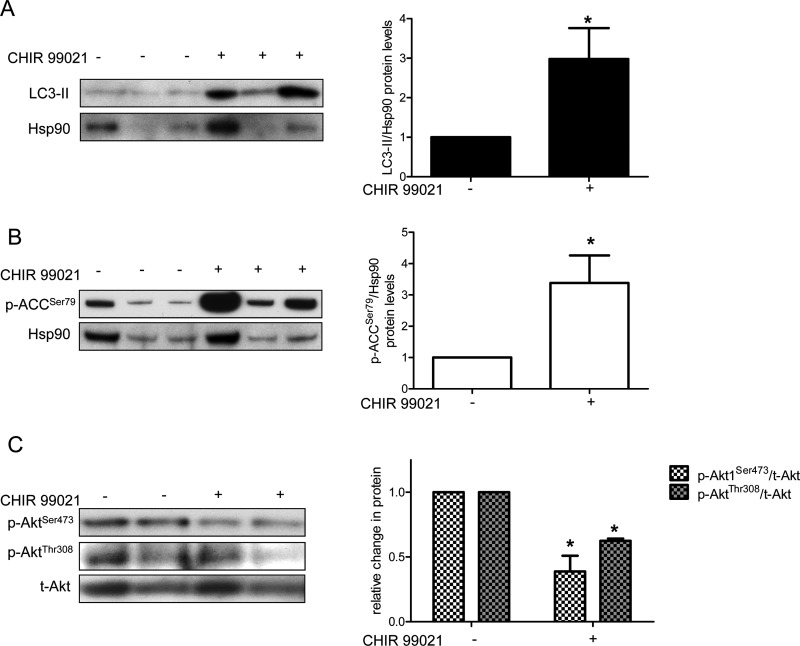
CHIR 99021 increases LC3-II protein levels and AMPK signalling and decreases Akt signalling in the mouse aorta Compared with aortas from control-treated mice (injected with saline/PEG400), aortas from mice treated with CHIR 99021 had higher levels of LC3-II protein (**A**), a marker of autophagosomes and p-ACC^Ser79^ (**B**), a downstream target of AMPK. CHIR 99021-treated aortas also had decreased ratios of p-Akt1^Ser473^/t-Akt and p-Akt^Thr308^/t-Akt (**C**). For (**A**) and (**B**), the same eight samples were run on two different gels to optimize visualization of each protein band. Densitometry is shown adjacent to each blot. * indicates *P*<0.05 (*n*=8).

To validate our studies in HAECs, and explore the efficacy of regulating autophagy via GSK3β inhibition *in vivo*, we treated mice with 7.5 mg/kg per day CHIR 99021 for 3 days to inhibit GSK3β activity in the aorta (Supplementary Figure S8). Similar to our findings in shGSK3β-infected HAECs (Figures 4A, Supplementary Figure S5A), inhibition of GSK3β with CHIR 99021 increased protein levels of LC3-II in the mouse aorta (Figure 7A). CHIR 99021 also increased AMPK activity (as indicated by an increase in protein levels of p-ACC^Ser79^) (Figure 7B). We also observed decreased ratios of p-Akt1^Ser473^/t-Akt and p-Akt^Thr308^/t-Akt in the aortas of CHIR 99021-treated mice (Figure 7C), suggesting that *in vivo*, GSK3β inhibition may affect similar signalling pathways as elucidated in human primary cell culture [30,57–67].

## DISCUSSION

**Figure 8:**
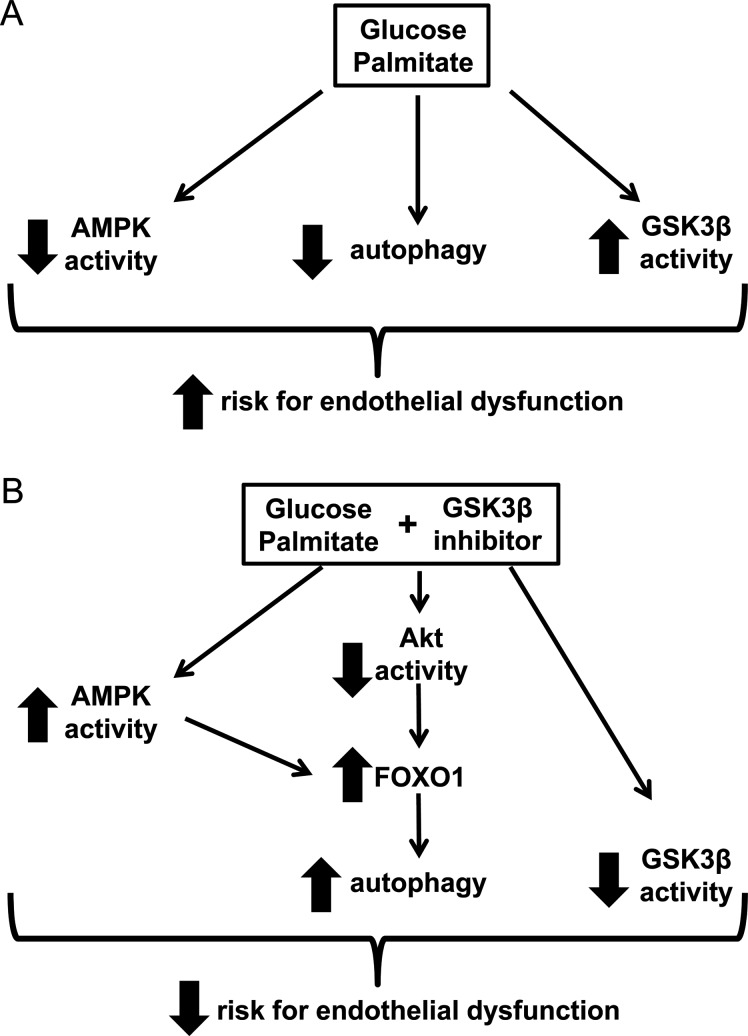
Potential use of GSK3β inhibitors to reduce excess nutrient-induced endothelial dysfunction (**A**) In the presence of high concentrations of glucose and palmitate, AMPK activity and basal autophagy decrease whereas GSK3β activity increases. These changes predispose endothelial cells to atherogenesis by increasing risk for endothelial dysfunction. (**B**) Inhibition of GSK3β in the presence of excess nutrients increases AMPK activity and autophagy, thereby reducing risk for endothelial dysfunction.

We previously showed that high concentrations of glucose and palmitate decrease AMPK activity and basal autophagy in HAECs [1]. In this work, we pursued the mechanism by which this occurs and found that it involves GSK3β (Figure 8A). Consistent with reports in other types of cells, we observed that GSK3β activity was inversely related to lysosome acidification in HAECs. Beyond this observation, our studies include the novel findings that (1) excess nutrients increase GSK3β activity, (2) increased GSK3β activity likely contributes to excess nutrient-induced decreases in lysosome acidification, (3) GSK3β activity is inversely related to autophagosome formation and unlike AMPK activation, GSK3β inhibition is an effective strategy to increase the number of autophagosomes under both control and excess nutrient conditions, (4) inhibition of GSK3β may induce autophagosome formation by activating FOXO1, potentially through increasing AMPK activity and/or decreasing Akt activity and (5) treatment of mice with the GSK3β inhibitor CHIR 99021 increases LC3-II protein levels and AMPK activity and decreases Akt activity in the mouse aorta, suggesting that targeting GSK3β may be a viable strategy to induce autophagy *in vivo*. Low levels of AMPK activity and autophagy in the presence of excess nutrients are associated with atherogenesis. Therefore, amplification of both of these processes by suppressing GSK3β may potentially reduce endothelial dysfunction (Figure 8B).

Impaired lysosome acidification has been inversely related to GSK3β activity in a number of different cell types, although the mechanism underlying this relationship seems to vary. In SHSY-5Y cells, GSK3β inhibitor-induced increases in lysosomal acidification were attributed to glycosylation of the lysosomal v-ATPase [23], whereas in HEK293 cells, they were associated with increased nuclear translocation of TFEB [25], a transcription factor for autophagy-related genes. In our HAEC model, GSK3β may be modifying the v-ATPase, but we doubt that TFEB is involved since GSK3β inhibition did not affect lysosome abundance (Figure 2B).

In addition to GSK3β, changes in AMPK activity in our HAEC model of type 2 diabetes could contribute to alterations in lysosome acidification. In rat microglia, activation of AMPK by metformin decreased lysosomal pH [26] suggesting that active AMPK may stabilize the lysosome. Since inhibition of GSK3β in HAECs not only attenuated the effect of excess nutrients on lysosome acidification (Figure 2A), but increased AMPK activity in excess nutrient-exposed cells (Figure 6B), AMPK may be mediating the effects of GSK3β on the lysosome. Alternatively, GSK3β-induced damage to the lysosome may contribute to the decreased AMPK activity that we previously observed in our excess nutrient-exposed HAECs [1]. AMPK can be activated at the lysosome [40,68], and inhibition of lysosome acidification has been shown to suppress AMPK activity in several cancer cell lines [69]. These speculations require further inquiry, as it is unknown how AMPK and the lysosome interact under excess nutrient conditions and whether this interaction involves GSK3β.

The majority of studies that relate GSK3β with autophagy do so through its effects on the lysosome. We found that in addition to the lysosome, GSK3β activity can also modulate autophagosome formation, perhaps through Akt/AMPK-FOXO1 signalling. Future work will not only clarify the key molecules involved in this signalling cascade, but also whether FOXO1 increases autophagy in our shGSK3β-infected HAECs via increased gene expression of *PIK3C3* and *Sod2* or through increased association with Atg7. It is also possible that FOXO1-independent factors could be involved. For example, suppression of Akt phosphorylation in shGSK3β-infected cells could promote autophagosome formation by reducing phosphorylation of beclin-1 [70].

Our observation of decreased Akt activity in shGSK3β-infected cells is distinct from Akt's established role as a kinase upstream of GSK3β. We suspect that this inhibition of Akt activity may be due to changes in phosphoinositide-3 kinase (PI3K) localization. It has been shown in *dictyostelium* that GSK3 phosphorylates PI3K1 and that this phosphorylation is required for its cAMP-induced membrane localization [71]. Membrane-localized PI3K1 is crucial for its downstream signalling, as genetic ablation of GSK3 in *dictyostelium* decreases protein kinase B (Akt) phosphorylation [72]. Although these relationships need to be verified in our model, these data suggest that inhibition of GSK3β may prevent PI3K from migrating to the membrane and thus suppress Akt activity. We speculate that this negative feedback loop may exist to prevent excessive suppression of GSK3β activity.

The primary objective of these studies was to understand the downstream consequences of GSK3β inhibition, in order to determine if it would be a suitable target for modulation of autophagy in cells exposed to excess nutrients. However, future work will elucidate how GSK3β is initially affected by high concentrations of glucose and palmitate. There are several possible molecules which might activate GSK3β in the presence of excess nutrients, including Akt [30], protein phosphatase 2A (PP2A) [57–60] and Wnt [61]. Since we did not observe any change in the ratio of p-Akt1^Ser473^/t-Akt between control and excess nutrient conditions (Supplementary Figure S9), we will focus our studies on PP2A, a molecule whose activation has been observed in animal models of insulin resistance [62,63] and in cell-culture studies using high concentrations of glucose and palmitate [64–66]. Our previous work in HUVECs indicated that the same concentrations of glucose and palmitate used in the current study increased cellular concentrations of ceramide, an activator of PP2A. Furthermore, we found that preventing nutrient-induced ceramide accumulation, through the overexpression of the lysosomal enzyme acid ceramidase, attenuated nutrient-induced losses in phosphorylation of uncoordinated 51-like kinase 1 [1], a kinase involved in autophagosome formation. In addition to activating GSK3β, PP2A is also associated with reduced AMPK activity [64]. Thus, nutrient-induced PP2A activation that increases GSK3β activity in our model would be consistent with two inverse associations that we observed in the present study: (1) between GSK3β and autophagosome formation (Figure 4) and (2) between GSK3β and AMPK signalling (Figure 6).

Preliminary findings from our laboratory indicate that treatment of HAECs with the PP2A inhibitor okadaic acid [60,67] inhibited GSK3β activity and increased AMPK activity under control conditions (Supplementary Figure S10A). Similar changes were observed under excess nutrient conditions (Supplementary Figure S10B), although the effect of okadaic acid was not as robust. This may be due to a higher amount of residual PP2A activity under excess nutrient conditions following okadaic acid treatment. Nevertheless, these data suggest that in the presence of excess nutrients, inhibition of PP2A can reduce GSK3β activity. We also found that in the presence of excess nutrients, okadaic acid increased both the level of bafilomycin-induced LC3-II protein as well as the LC3-II/LC3-I ratio (Supplementary Figure S11A), but did not alter lysotracker staining (Supplementary Figure S11B). Together, these data suggest that in addition to activating GSK3β, excess nutrient-induced changes in PP2A may alter autophagosome formation, but are not alone sufficient to affect lysosome acidification. Thus, exposure of HAECs to excess nutrients may activate several factors, including PP2A, which activate GSK3β and subsequently affect different elements of the autophagy process (Supplementary Figure S11C).

PP2A has been shown to activate GSK3β activity in cancer cells as well as neurons [57–60,73]. Interestingly in HEK293 cells, not only does inhibition of PP2A inhibit GSK3β, but overexpression of GSK3β increases PP2A activity [64,73,74–76]. An inverse relationship between PP2A and autophagy has been found in MCF-7 cells [77], cortical neurons [78], rat neurons [79] and rat hepatocytes [80]. However, other studies (using similar doses of okadaic acid) in cortical neurons [81] and rat hepatocytes [82,83] have suggested that PP2A inhibition inhibits autophagy. This lack of consensus in the literature may be due to heterogeneity in treatment conditions among these studies. Since there are likely to be a large number of PP2A substrates, differences in nutrient status and okadaic acid treatment duration may affect several PP2A substrates that differentially affect autophagy. Clearly, much remains to be discovered regarding the actions of PP2A, including whether GSK3β is activating PP2A in excess nutrient-exposed HAECs and whether induction of autophagy by PP2A inhibition requires modulation of GSK3β activity.

GSK3β inhibitors have been associated with therapeutic benefits for a number of conditions associated with hyperglycaemia and dyslipidaemia [20,84–89]. Experimental human and animal models have shown that GSK3β inhibitors decrease blood glucose levels and improve insulin sensitivity [89–92], perhaps by reducing phosphorylation of IRS-1 (to increase IRS-1 stability and improve insulin action) [89,93,94] and/or reducing phosphorylation of GS (to increase GS activity and the conversion of glucose to glycogen) [90,91]. Increased glucose transport upon GSK3β inhibition has also been associated with increased GLUT4 expression in muscle, increased phosphorylated AMPK in the liver and decreased GLUT2 expression in the liver [89]. In addition to these actions on metabolism, GSK3β inhibitors have been associated with reduction of diabetic complications in the heart [20,84], vasculature [86–88] and pancreas [89]. These complications have been associated with lipid accumulation, inflammation, fibrosis, oxidative damage and endoplasmic reticulum stress, all of which are reduced by GSK3β inhibition possibly through mediators such as sterol regulatory binding protein (SREBP), caspase 3 and NF-κB [20,86,89]. Studies in rat cerebral microvascular endothelial cells suggest that suppression of Wnt signalling may also link GSK3β hyperactivation with diabetic vascular damage. Chong and colleagues found that subjecting these cells to hyperglycaemic conditions increased GSK3β activity as well as apoptosis [95,96]. Treatment with erythropoietin improved cell survival in hyperglycaemia by stabilizing Wnt and activating Akt, thereby inhibiting GSK3β and stabilizing β-catenin [96]. Our work expands upon this body of literature, showing that GSK3β inhibition may attenuate diabetes-induced autophagy impairment in HAECs by increasing autophagosome formation, potentially by Akt/AMPK-FOXO1 signalling. Our analysis of autophagy and its related signalling in the mouse aorta lays the foundation for future investigations of GSK3β and autophagy in the aorta and more specifically, aortic endothelial cells, in mouse models of diabetes. These studies will help not only clarify the role of autophagy in diabetic vascular disease, but also elucidate the relative contribution of autophagy to the effects of GSK3β inhibitors.

Despite this growing body of preclinical data supporting a role for GSK3β in diabetes, most clinical trials using GSK3β inhibitors study neurological diseases such as Alzheimer's Disease and Progressive Supranuclear Palsy [97,98]. One potential way to improve the efficacy of GSK3β inhibitors in metabolic disease (and potentially prompt future clinical trials) could be to include them in combination therapies. For example, combining a therapy that inhibits GSK3β (to increase autophagy) with one that activates AMPK (to reduce inflammation and oxidative stress) may improve the prognosis for diabetic cardiovascular complications [68].

Collectively, we have shown that the conditions which mimic those of type 2 diabetes increased GSK3β activity in HAECs. Suppression of GSK3β in this nutrient-laden environment not only increased AMPK signalling but also increased certain features of basal autophagy. These effects on basal autophagy may be dependent on FOXO1, in concert with Akt and/or AMPK signalling. By unravelling more of the mechanistic details by which high concentrations of glucose and palmitate impair basal autophagy, this work brings us one step closer to the development of therapies that maintain vascular endothelial cell health amidst diabetic stresses.

## Abbreviations

ACC: acetyl-CoA carboxylase
AMPK: AMP-activated protein kinase
FOXO1: forkhead box protein O1
GSK3β: glycogen synthase kinase 3β
HAEC: human aortic endothelial cell
HUVEC: human umbilical vein endothelial cell
LC3: microtubule-associated protein light chain 3
MEF: mouse embryonic fibroblast
mTORC1: mammalian target of rapamycin complex 1
PP2A: protein phosphatase 2A
TFEB: transcription factor EB
